# Three new species of *Osmylus* Latreille from China (Neuroptera, Osmylidae)

**DOI:** 10.3897/zookeys.589.7320

**Published:** 2016-05-16

**Authors:** Han Xu, Yongjie Wang, Zhiqi Liu

**Affiliations:** 1 Department of Entomology, China Agricultural University, Beijing 100094, China; 2 College of Life Sciences, Capital Normal University, Beijing 100048, China

**Keywords:** New species, Oriental region, Osmylidae, Osmylus, Palaearctic region

## Abstract

Three new species of *Osmylus* Latreille are described from China: *Osmylus maoershanicola* **sp. n.**, *Osmylus shaanxiensis* **sp. n.** and *Osmylus angustimarginatus* **sp. n.** These new species are distinguishable from other related species by the shape of the 9^th^ tergite of both sexes, as well as the shape of gonarcus, mediuncus and spermatheca. A key is given to differentiate Palaearctic and Oriental species of *Osmylus*.

## Introduction

**Figure 1.**
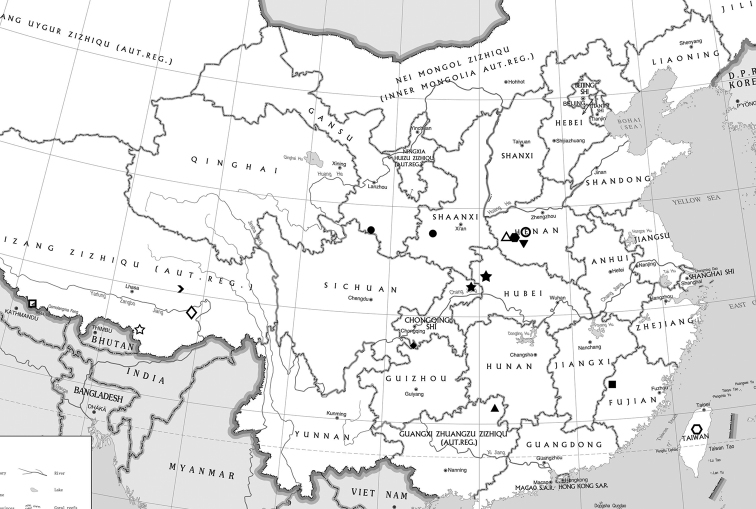
Distribution of *Osmylus* in China. ◆ = *Osmylus angustimarginatus* sp. n.; △ = *Osmylus biangulus*; ▼ = *Osmylus bipapillatus*; ☆ = *Osmylus conanus*; ★ = *Osmylus fuberosus*; ⬣ = *Osmylus lucalatus*; ▲ = *Osmylus maoershanicola* sp. n.; ◇ = *Osmylus megistus*; □ = *Osmylus minisculus*; ○ = *Osmylus shaanxiensis* sp. n.; ⬡ = *Osmylus taiwanensis*; ■ = *Osmylus wuyishanus*; > = *Osmylus xizangensis*.

The genus *Osmylus* Latreille (Osmylidae: Osmylinae) contains 21 species distributed in the Palaearctic and Oriental regions, 20 species of which are distributed in Asia and only one, *Osmylus fulvicephalus*, which is widespread in Europe (Banks 1947, Canbulat 2013, Iwata 1928, Kozhanchikov 1951, Krüger 1912, 1913, Makarkin 1985, McLachlan 1870, 1875, Nakahara 1914, New 1991, Yang 1987, 1988, 1997, 1999). The first *Osmylus* species of the Chinese fauna, Osmylus (Lysmus) oberthurinus, was described by Navás (1910) and then 12 species described successively by Banks (1947), Yang (1987, 1988, 1997, 1999) and Wang and Liu (2010), with higher diversity in Tibet (four species) and Shaanxi Province (four species) (Fig. 1).

The biology of osmylids is still poorly known. *Osmylus* is known to be univoltine and adults feed as generalists on fungal spores, pollen, algae, mites and insects; they sit temporarily on foliage of plants along streams or river banks in daytime (Devetak 2007, Gepp 1976, Withycombe 1923). The biology of larvae remains controversial. Latreille (1805) and Stein (1838) deduced that the larvae of *Osmylus* are aquatic. However, Stitz (1936) and Eisner (1989) reported correctly that the larvae simply live in the water margin (the riparian interface) but cannot survive submersion. Accordingly, the larvae should definitely be regarded as terrestrial.

## Materials and methods

The specimens in this study were examined under an Optec SZ760 stereomicroscope with direct light. The terminal of abdomens were removed and soaked in the 10% NaOH for boiling water bath and stored in a glycerin-filled micro-vial mounted on the pin beneath specimen. The terminology for wing venation and genitalia follows New (1983), Adams (1969) and New (1983). All type specimens are deposited in the Entomological Museum of China Agricultural University
(CAU), Beijing.

## Taxonomy

### Genus Osmylus

Taxon classificationAnimaliaNeuropteraOsmylidae

Latreille

Osmylus Latreille, 1802: 289. Type species: Hemerobius fulvicephalus Scopoli, 1763: 270.
Dictyosmylus Navás, 1910: 189. Type species: Dictyosmylus lunatus Navás, 1910: 189, by monotypy.

#### Diagnosis.

Moderate to large body size (body length 15–20 mm); forewing generally large and broad (length 20–30 mm), with numerous fragmentary marks; two nygmata present at the center and the proximal base of wing between MP and Rs; veins dark brown; costal cross-veins generally bifurcate distally, without interlinking veinlets; cross-veins among branches of Rs forming at least two series of gradates; MP forked close to the base, MP_2_ with many branches. The hindwing resembles the forewing in shape, but with fewer spots. The 9^th^ tergite has variably-shaped dorsal process. Genitalia are composed of a gonarcus and a mediuncus; the gonarcus is variable in shape, consisting of a sclerotized and pilose external section posteriorly with a lightly sclerotized anterior-lateral section, the latter laterally with an anterior rod shaped process (i.e., baculum of some authors) which is sometimes articulated. The mediuncus (i.e., parameres of some authors) is curved with a fused base (although the shape is variable in *Osmylus pachycaudatus*). The mediuncus is subtended laterally by the rod-shaped, paired parameres (i.e., subarcus of other authors) that are not fused anteriorly. The female 9^th^ tergite occasionally has a ventral process, the gonapophysis lateralis is generally finger-like and articulated with stylus distally, and the spermatheca is either oval or cylindrical in shape.

#### Included species.

*Osmylus angustimarginatus* sp. n., *Osmylus biangulus* Wang & Liu, *Osmylus bipapillatus* Wang & Liu, *Osmylus cilicicus* Krüger, *Osmylus conanus* Yang, *Osmylus decoratus* Nakahara, *Osmylus fuberosus* Yang, *Osmylus fulvicephalus* (Scopoli), *Osmylus gussakovskii* Kozhanchikov, *Osmylus hyalinatus* McLachlan, *Osmylus kisoensis* Iwata, *Osmylus lucalatus* Wang, *Osmylus maoershanicola* sp. n., *Osmylus megistus* Yang, *Osmylus minisculus* Yang, *Osmylus multiguttatus* McLachlan, *Osmylus pachycaudatus* Wang, *Osmylus posticatus* Banks, *Osmylus pryeri* McLachlan, *Osmylus shaanxiensis* sp. n., *Osmylus taiwanensis* New, *Osmylus tessellatus* McLachlan, *Osmylus wuyishanus* Yang, *Osmylus xizangensis* Yang.

#### Comments.

*Osmylus* has been often confused with three other genera, *Grandosmylus* Makarkin, 1985, *Parosmylus* Needham, 1909 and *Plethosmylus* Krüger, 1913. Banks (1913) advanced that *Parosmylus* should be a junior synonym of *Osmylus* because the spur on the coxa in *Parosmylus* is also present in some species of *Osmylus*. Krüger (1913) erected the genus *Plethosmylus* based on venation characters (presence of interlink veinlets between costal cross-veins). Nakahara (1914) considered the opinion of Krüger subjective and synonymized the latter genus. Kuwayama (1953, 1962) again separated *Plethosmylus*, differentiating it from *Osmylus* by the presence of interlinking veinlets among the costal and two basal Rs-Mp cross-veins before the proximal nygma. However, Makarkin (1985) revised the status of *Plethosmylus*, synonymizing it with *Osmylus* and establishing a new subgenus *Plesiosmylus* within *Osmylus*. He also established a new genus *Grandosmylus*, separated from *Osmylus* by the irregular gradate cross-veins and the shape of 9^th^ sternite in males and 8^th^ sternite in females; this opinion was accepted by Sekimoto (2011) in his revision of Japanese *Osmylus*. The relationship among *Grandosmylus*, *Parosmylus* and *Plethosmylus* remains unclear. Wang and Liu (2009) clarified the generic status of *Parosmylus*, after reviewing specimens from mainland China, and they concluded that both genera could be valid due to differences in the number of gradate series, the configuration of gonarcus and the shape of spermatheca (Wang and Liu 2009). Furthermore, after re-examining the specimens of *Plethosmylus* from mainland China, we observed that *Osmylus* and *Plethosmylus* possessed significant differences in male genitalia (the configuration of gonarcus) and in female genitalia. Moreover, the interlink veinlets among costal cross-veins could conveniently divide them. Considering the vague relationships among these genera, we consider is suitable to maintain them as separate genera until a robust phylogenetic work can be conducted in the future. In this paper, three new species of *Osmylus* are described from China: *Osmylus maoershanicola* sp. n. *Osmylus shaanxiensis* sp. n. and *Osmylus angustimarginatus* sp. n., primarily based on genital characters.

#### Key to *Osmylus* species in the Palaearctic and Oriental regions

(Note: *Osmylus kisoensis* is not included as it is only known from the larval stage, while *Osmylus cilicicius* and *Osmylus posticatus* are poorly known and could not be included in the key.)

**Table:** 

1	The structure of spermatheca complicated (Fig. 2a)	***Osmylus megistus***
–	The structure of spermatheca simple (Fig. 2b–h)	**2**
2	The 7^th^ sternite in female with a median preapical protuberance	**3**
–	The 7^th^ sternite in female without any protuberance	**4**
3	Spermatheca cylindrical and bent; anterior third of pronotum with median stripe	***Osmylus taiwanensis***
–	Spermatheca oval; frons with dark brown X-shaped marking; pronotum with yellow and median stripe	***Osmylus decoratus***
4	The gonapophyses lateralis cone-shaped, spermatheca pyriform	***Osmylus minisculus***
–	The gonapophyses lateralis finger-like or fusiform	**5**
5	9^th^ tergite in male with a distinct dorsal process (Figs 3a–d, 5a, 9a)	**6**
–	9^th^ tergite in male without distinct dorsal processes (Figs 3e, 7a)	**14**
6	Gonarcus with a sharpened process along dorsal margin in lateral view	***Osmylus pryeri***
–	Gonarcus without processes along dorsal margin in lateral view	**7**
7	Forewing relatively narrow, membrane hyaline with slight metallic luster	***Osmylus lucalatus***
–	Forewing broad, membrane dull hyaline	**8**
8	The length of process of 9^th^ tergite in male slightly longer than width (Figs 3b, 5a, 9a)	**9**
–	The length of process of 9^th^ tergite in male significantly longer than width (Fig. 3c–d)	**13**
9	The process of 9^th^ tergite in male cone-shaped	***Osmylus hyalinatus***
–	The process of 9^th^ tergite in male bar-shaped	**10**
10	Gonarcus with one or two lateral posteroventral protuberances	**11**
–	Gonarcus without any lateral posteroventral protuberance	**12**
11	Gonarcus with only one lateral posteroventral protuberance in lateral view (Fig. 5b)	***Osmylus maoershanicola* sp. n.**
–	Gonarcus with two lateral posteroventral protuberances in lateral view (Fig. 3b)	***Osmylus biangulus***
12	Mediuncus apically hook-shaped	***Osmylus fulvicephalus***
–	Mediuncus apically slender and straight (Fig. 5d)	***Osmylus angustimarginatus* sp. n.**
13	The process of 9^th^ tergite in male cylindrical (Fig. 3c), mediuncus arch-like in lateral view, anterior arm of gonarcus with a distal right-angle bend	***Osmylus pachycaudatus***
–	The process of 9^th^ tergite in male subulate (Fig. 3d), mediuncus C-shaped in lateral view	***Osmylus bipapillatus***
14	Distal part of gonarcus flat and quadrate, with a posteroventral protuberance (Fig. 3e)	***Osmylus fuberosus***
–	Distal part of gonarcus approximately triangular	**15**
15	Distal part of gonarcus with one or more conspicuous protuberances	**16**
–	Distal part of gonarcus with an inconspicuous protuberance	**20**
16	8^th^ sternite in female with two ventral protuberances; distal part of gonarcus with a ventral and rod-like protuberance	***Osmylus gussakovskii***
–	8^th^ sternite in female without any protuberance	**17**
17	Distal part of gonarcus with two protuberances in later view; pronotum with slender yellow marking on anterior half and yellow spot over posterior margin	***Osmylus tessellatus***
–	Distal part of gonarcus with only one protuberance	**18**
18	Cross-veins among branches of Rs forming 3 series of gradates; forewing and hindwing with approximately rounded spots	***Osmylus multiguttatus***
–	Cross-veins among branches of Rs forming 2 series of gradates	**19**
19	9^th^ tergite in female with a median narrowing in lateral view (Fig. 7e); meso- and metanotum dark brown, some sclerites brown	***Osmylus shaanxiensis* sp. n.**
–	9^th^ tergite in female slightly tapered medially in lateral view; meso- and metanotum yellowish brown with black streaks	***Osmylus xizangensis***
20	Distal part of gonarcus forming a large triangular sclerite; outer gradates of forewing with brown marks	***Osmylus wuyishanus***
–	Distal part of gonarcus forming a narrow and small sclerite; inner gradates of forewing with brown marks	***Osmylus conacus***

**Figure 2.**
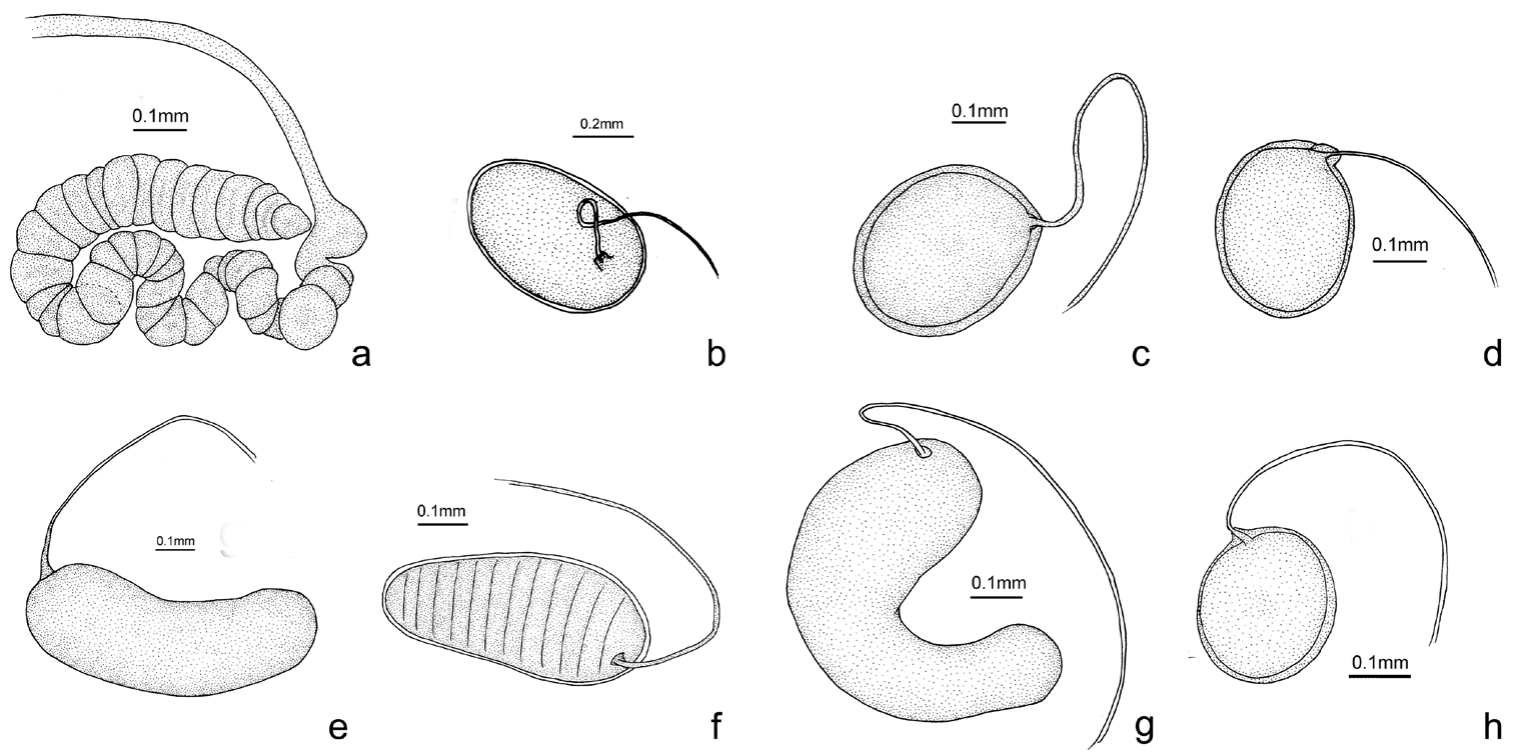
Spermathecae. **a** *Osmylus megistus* **b** *Osmylus lucalatus* **c** *Osmylus angustimarginatus* sp. n. **d** *Osmylus maoershanicola* sp. n. **e** *Osmylus biangulus* **f** *Osmylus fuberosus* **g** *Osmylus shaanxiensis* sp. n. **h** *Osmylus wuyishanus*.

**Figure 3.**
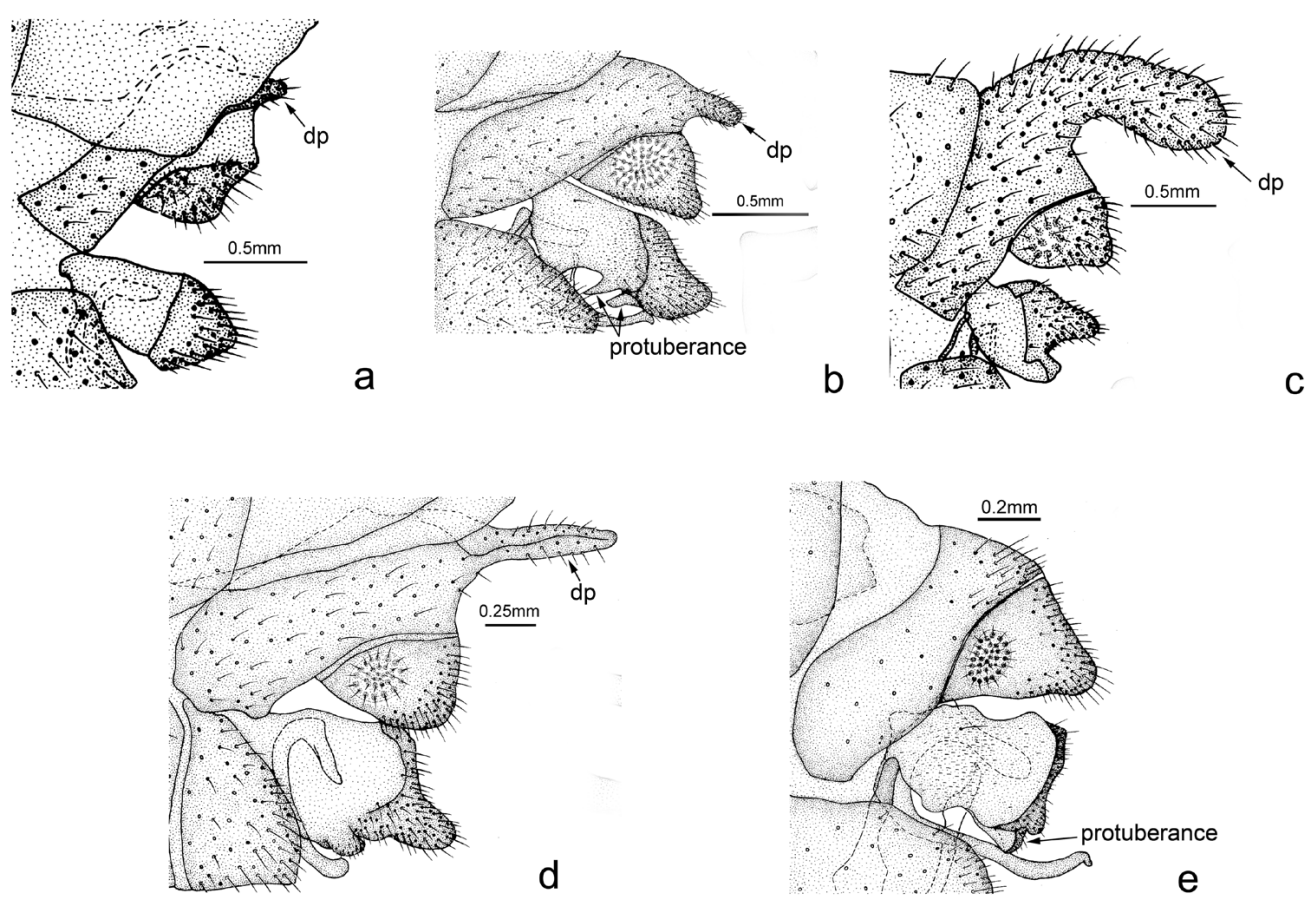
Male terminalia, lateral view. **a** *Osmylus lucalatus* **b** *Osmylus biangulus* **c** *Osmylus pachycaudatus* **d** *Osmylus bipapillatus* **e** *Osmylus fuberosus*. Abbreviation: dp, dorsal process.

**Figure 4.**
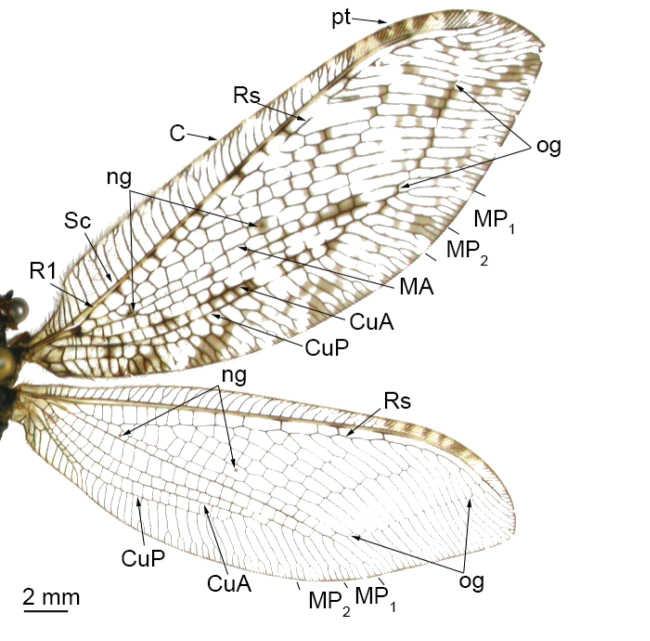
Wings of *Osmylus maoershanicola* sp. n., forewing (upper) and hindwing (lower). Abbreviations: ng, nygmata; pt, pterostigma; og, outer gradates.

**Figure 5.**
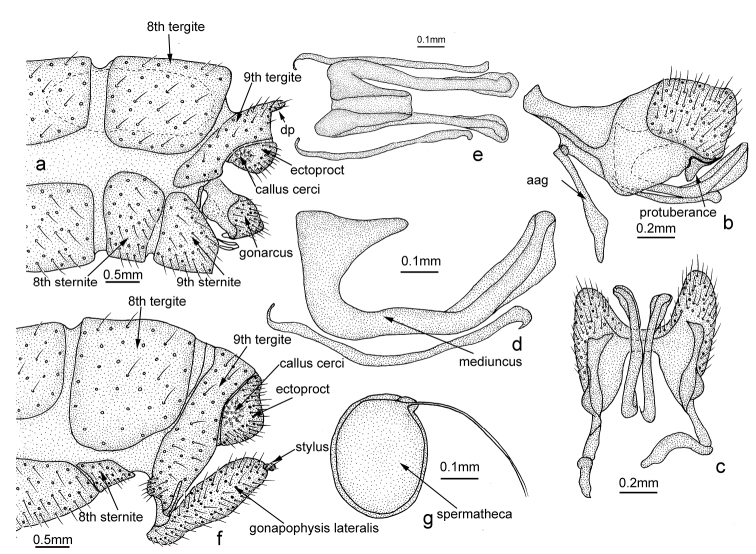
*Osmylus maoershanicola* sp. n. **a–e** Male: **a** apex of the abdomen and genitalia, lateral view **b** genitalia, lateral view **c** genitalia, ventral view **d** mediuncus, lateral view **e** mediuncus, dorsal view **f–g** Female: **f** apex of the abdomen and genitalia **g** spermatheca, lateral view (spiracula omitted). Abbreviation: dp, dorsal process; aag, anterior arm of gonarcus.

**Figure 6.**
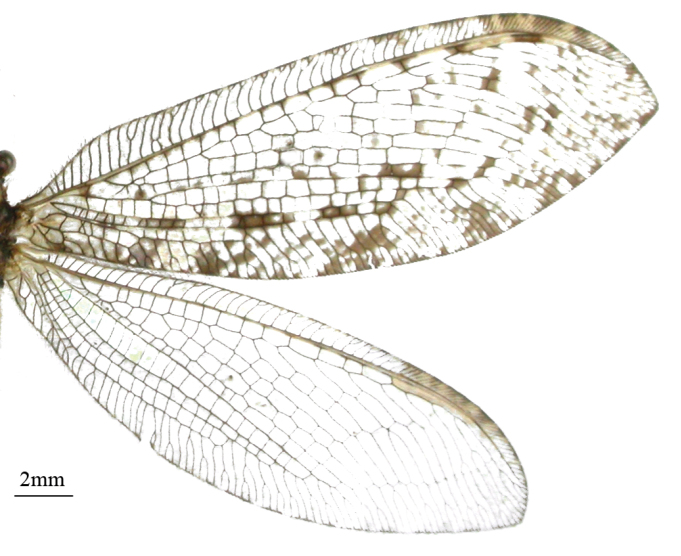
Wings of *Osmylus shaanxiensis* sp. n., forewing (upper) and hindwing (lower).

**Figures 7.**
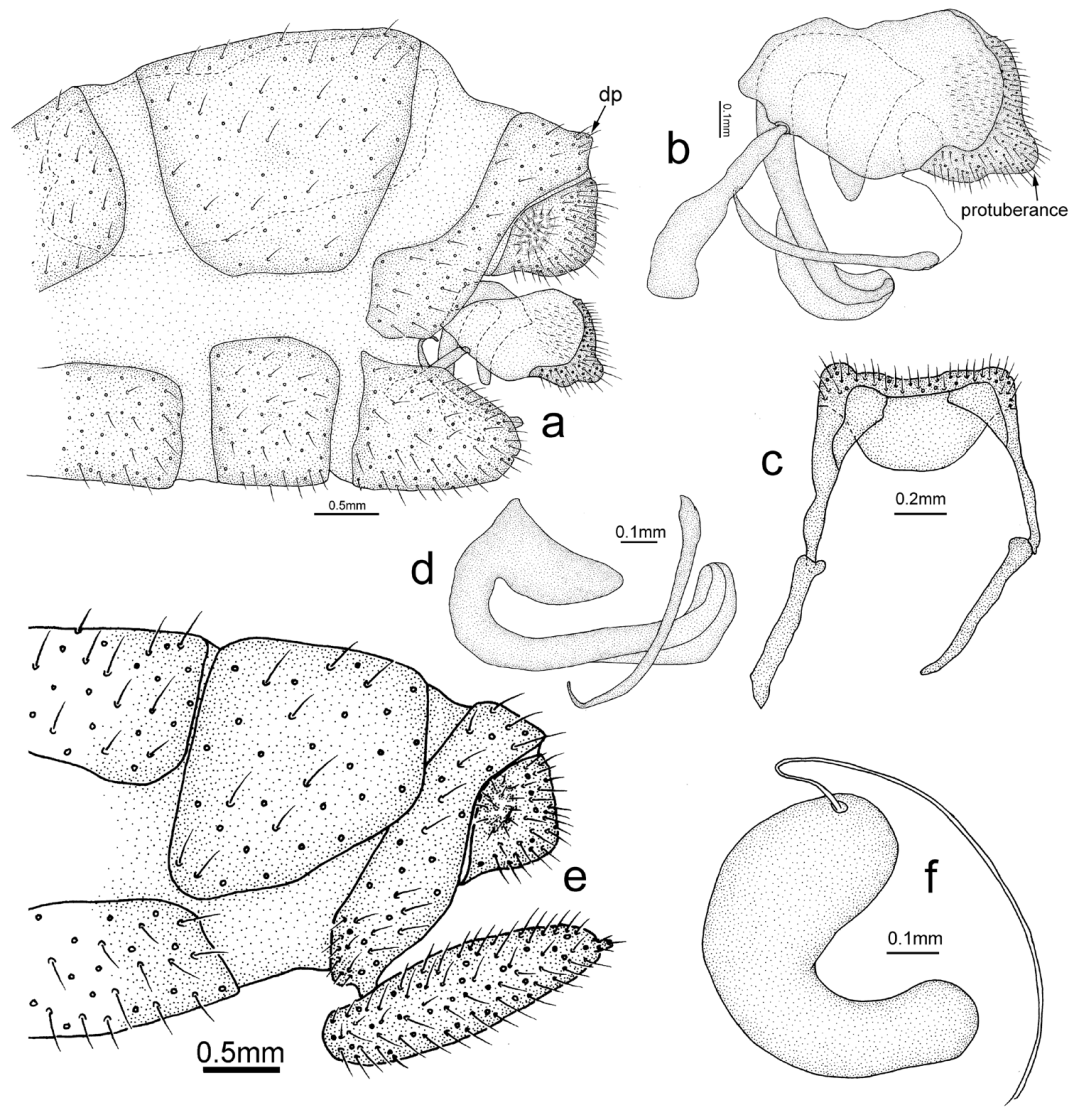
*Osmylus shaanxiensis* sp. n. **a–d** Male: **a** apex of the abdomen and genitalia **b** genitalia, lateral view **c** genitalia, ventral view **d** mediuncus, lateral view **e–f** Female: **e** apex of the abdomen and genitalia **f** spermatheca, lateral view. Abbreviation: dp, dorsal process.

**Figure 8.**
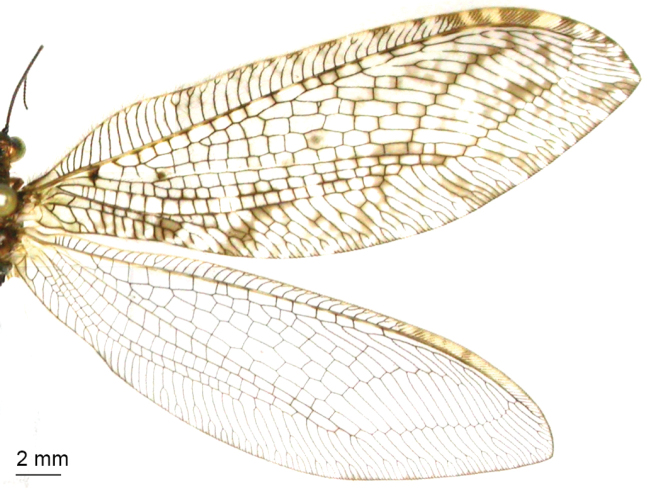
Wings of *Osmylus angustimarginatus* sp. n., forewing (upper) and hindwing (lower).

**Figure 9.**
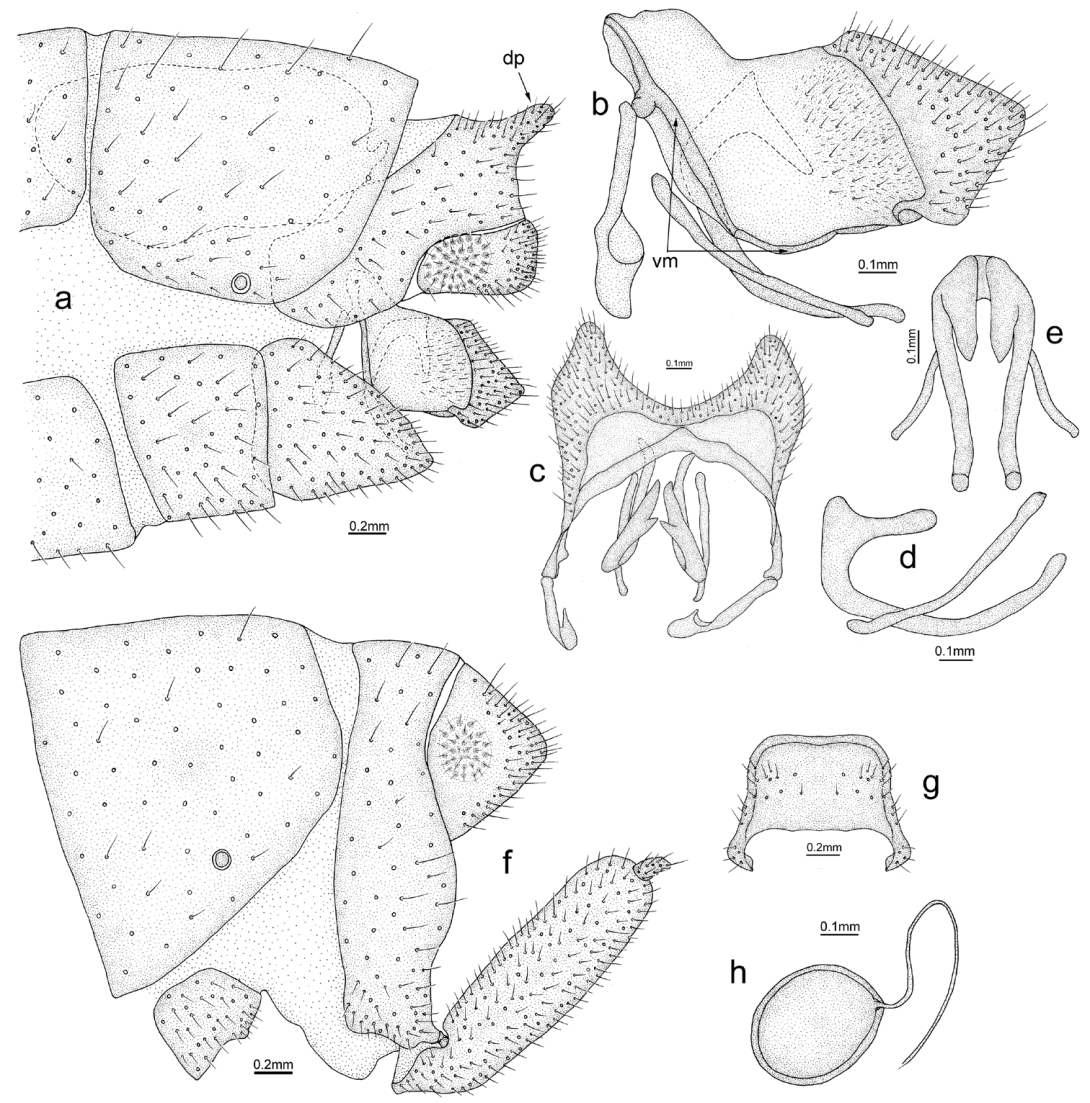
*Osmylus angustimarginatus* sp. n. **a–e** Male: **a** apex of the abdomen and genitalia **b** genitalia, lateral view **c** genitalia, dorsal view **d** mediuncus, lateral view **e** mediuncus, dorsal view **f–h** Female: **f** apex of the abdomen and genitalia **g** subgenital plate, ventral view **h** spermatheca, lateral view. Abbreviations: dp, dorsal process; vm, ventral margin.

### Osmylus maoershanicola sp. n.

Taxon classificationAnimaliaNeuropteraOsmylidae

http://zoobank.org/515687EC-B4FF-489C-A12D-4B41DEC81A34

Figs 4 , 5

#### Material examined.

Holotype Male, CHINA: Guangxi (Province): Maoershan (Nature Reserve), [25°48'N, 110°24'E], 9.viii.2005, leg. Ping Zhao. Verbatim label data (translated from Chinese): CHINA: Guangxi Prov., Maoershan/ 9.viii.2005/ Ping Zhao/ CAU. Condition: Antennal flagellum missing. Abdomen terminalia cleared in KOH, and stored in the micro-vial pinned below the specimen. Paratype. 1 female (left antenna damaged), same data as holotype (CAU).

#### Diagnosis.

Male: 9^th^ tergite with a short finger-like dorsal process; ectoproct cone-shaped. Gonarcus distally triangular with a ventral, triangular, membranous protuberance in lateral view. Female: gonapophysis lateralis approximately fusiform; spermatheca oval.

#### Description.

*Head*. Vertex yellowish-brown with brown setae; eye dark gray, ocelli yellow, area within ocelli black. Antennal flagellum missing, scape and pedicel dark brown; frons yellow. *Thorax*. Pronotum dark brown, posterior margin slightly wider, with black brown setae; meso- and metanotum black with brown setae. Legs yellow with brown setae; pretarsal claws dark brown.

*Wing* (Fig. 4). Forewing length 27–28 mm, width 9–10 mm. Membrane hyaline, with many sparse, fuscous spots; pterostigma brown; nygmata light brown; veins dark brown; Rs with 13–14 branches, outer gradate cross-veins edged with fuscous stains; R1-Rs cross-veins edged with brown marks; short cross-veins are present among the branches of CuP. Hindwing length 23–24 mm, width 7–8 mm. Membrane hyaline; pterostigma light yellow.

*Male terminalia* (Fig. 5a–e). Scent glands slender. 9^th^ tergite long and narrow with a short, dorsal finger-like process (Fig. 5a), ventral margin slightly tapered. 9^th^ sternite trapezoidal in lateral view. Ectoproct triangular in lateral view, callus cerci round. Distal part of gonarcus well sclerotized and approximately triangular, ventral part membranous with a triangular protuberance in lateral view (Fig. 5b); anterior arm of gonarcus slender; mediuncus dilated basally with a sharp backward end, slender apically and coated by a membrane in lateral view; rod-shaped paramere beneath the mediuncus slightly bent in lateral view, posterior end sharp.

*Female terminalia* (Fig. 5f–g). 8^th^ sternite approximately trapezoidal; 9^th^ tergite long and narrow with a ventral hemispherical tubercle in lateral view; ectoproct triangular in lateral view, callus cerci round, presenting in middle; gonapophysis lateralis approximately fusiform, stylus cylindrical; spermatheca simple, approximately spherical.

#### Distribution.

Presently known only from Guangxi Province, China.

#### Etymology.

The specific name ‘*maoershanicola*’ refers to ‘Maoershan Mountain’, the type locality.

#### Remarks.

The dorsal finger-like process of 9^th^ tergite of *Osmylus maoershanicola* sp. n. is similar to *Osmylus pryeri* and *Osmylus biangulus*, but this new species can be identified by the distinctive shape of the gonarcus. There are two prominent ventral protuberances in the distal part of gonarcus of *Osmylus pryeri* and *Osmylus biangulus* (Fig. 3b) but only one in *Osmylus maoershanicola* sp. n. (Fig. 5b). Furthermore, the distal gonarcus is cone-shaped in *Osmylus biangulus* but triangular in *Osmylus maoershanicola* and the spermatheca is short and bent rod-like in *Osmylus biangulus* (Fig. 2e) but approximately spherical in *Osmylus maoershanicola* (Fig. 5g).

### Osmylus shaanxiensis sp. n.

Taxon classificationAnimaliaNeuropteraOsmylidae

http://zoobank.org/0815CDFE-15C7-4C26-AC13-46F03D535A44

Figs 6 , 7

#### Material examined.

Holotype Male. CHINA: Shaanxi (Province): Houzhenzi (town), [33°51'N, 107°50'E] 12.viii.2007, leg. Yang Shi. Verbatim label data (translated from Chinese): CHINA: Shaanxi, Houzhenzi/ 12.viii.2007/ Yang Shi/ CAU. Condition: Antennal flagellum missing. Terminalia cleared in KOH, and stored in the micro-vial pinned below the specimen. Paratype. 1 female, same data as holotype (CAU). 1 female, CHINA: Gansu (Province): Diebu (county), Lazikou. 1700m, [34°03'N, 103°54'E] 12.viii.1980, Chikun Yang (CAU).

#### Diagnosis.

Wing broad, with numerous dark brown spots on the margin. Male: 9^th^ tergite with a median narrowing, with a small tuberous dorsal process in lateral view; protuberance of posteroventral gonarcus papillary. Base of mediuncus knife-shaped in lateral view. Female: gonapophysis lateralis basally fused with a triangular sclerite, spermatheca bent, cylindrical.

#### Description.

*Head*. Vertex dark brown. Ocelli yellow, area comprised among ocelli dark brown, eye dark brown; frons brown. *Thorax*. Pronotum dark brown with yellow long setae; meso- and metanotum dark brown. Legs yellow with dark yellow setae, pretarsal claws dark brown.

*Wings* (Fig. 6). Forewing length 22–25 mm, width 8–9 mm. Membrane hyaline, with numerous dark brown spots on the margin; pterostigma and nygmata brown; veins dark brown, some edged with dark brown spots; Rs with 12–13 branches, gradates cross-veins with brown marks. Hindwing length 20–22 mm, width 7–8 mm. Membrane hyaline; pterostigma light brown.

*Male Terminalia* (Fig. 7a–d). Scent glands stout; 9^th^ tergite with a median narrowing in lateral view, with a small hemispheric dorsal process; ectoproct triangular in lateral view, callus cerci oval; gonarcus sclerotized distally and posteroventrally ending into a papilla in lateral view; anterior arm of gonarcus slender, basally dilated; mediuncus basally dilated, knife-shaped, more slender apically in lateral view; rod-shaped paramere slender and bent in lateral view, dilating from base to end.

*Female Terminalia* (Fig. 7e–f). 8^th^ sternite reduced; 9^th^ tergite narrow; ectoproct approximately conical, callus cerci round; gonapophysis lateralis fusiform, apex with a long finger-like stylus; spermatheca cylindrical, bent and slightly dilated basally.

#### Distribution.

China (Shaanxi, Gansu).

#### Etymology.

The specific name ‘*shaanxiensis*’ refers to ‘Shaanxi Province’, the type locality.

#### Remarks.

The new species can be distinguished from other species by the small hemispheric dorsal process of the 9^th^ tergite in male (Fig. 7a). Although *Osmylus shaanxiensis* sp. n. is similar to *Osmylus conanus*, they can be easily separated by the differences of gonarcus and gonapophysis lateralis. The distal part of gonarcus in *Osmylus conanus* protrudes slightly but the same part in *Osmylus shaanxiensis* sp. n. protrudes significantly in lateral view (Fig. 7b). Also compared with *Osmylus conanus*, the spermatheca in *Osmylus shaanxiensis* sp. n. is longer and more bent (Fig. 7f).

### Osmylus angustimarginatus sp. n.

Taxon classificationAnimaliaNeuropteraOsmylidae

http://zoobank.org/8018E2FE-CCBB-4BD5-8D9D-B567A84A097A

Figs 8 , 9

#### Material examined.

Holotype Male. CHINA: Chongqing: Jiangjin (District): Simianshan (mountain), [28°38'N, 106°24'E] 17.vi.2006, leg. Weiwei Zhang. Verbatim label data (translated from Chinese): CHINA: Chongqing, Jiangjin, Simianshan/ 17.vi.2006/ Weiwei Zhang/ PC. Terminalia cleared in KOH, and stored in a micro-vial pinned below the specimen. Paratype. 1 female, same data as holotype; 1 male, 1 female, same locality as holotype. 21-23.ix.2007, leg. Weiwei Zhang.

#### Diagnosis.

Male: 9^th^ with a finger-like dorsal process. Gonarcus distally triangular in lateral view, ventral margin well sclerotized; base of mediuncus slightly protuberant distally in lateral view. Female: gonapophysis lateralis finger-like; spermatheca approximately spherical.

#### Description.

*Head*. Vertex yellow brown, with dark brown setae; ocelli light yellow, area comprised among ocelli dark brown; eyes gray with metallic reflection; frons black. *Thorax*. Pronotum dark brown, with yellow setae; meso- and metanotum fuscous, with black stripes. Legs yellow, with short setae, pretarsal claws dark brown.

*Wings* (Fig. 8). Forewing length 27–29 mm, width 8–9 mm. Wings elongated; membrane hyaline, with numerous brown spots; pterostigma brown, nygmata light brown; veins dark brown, some edged with dark brown spots; Rs with 13–14 branches; cross-veins are present among branches of CuP. Hindwing length 25–26 mm, width 7–8 mm; membrane hyaline; pterostigma light brown.

*Male Terminalia* (Fig. 9a–e). Scent glands stout. 9^th^ tergite wide, with a finger-like process; 9^th^ sternite approximately rectangular in lateral view; ectoproct small, callus cerci round; gonarcus distally well sclerotized and triangular in lateral view, ventral margin well sclerotized; anterior arm of gonarcus slender and basally dilated; mediuncus slightly finger-like at base, more slender apically in lateral view; rod-shaped paramere beneath the mediuncus slightly bent in lateral view.

*Female Terminalia* (Fig. 9f–h). 8^th^ sternite approximately square in lateral view. 9^th^ tergite narrow; ectoproct conical, callus cerci round; gonapophysis lateralis long and finger-like, with a long conical stylus; spermatheca approximately spherical.

#### Distribution.

Known only from Chongqing, China.

#### Etymology.

The specific name ‘*angustimarginatus*’ the compound of Latin deribation, from *angusti*- (narrow) and *marginatus*- (margin), refers to the well sclerotized ventral margin of the gonarcus in lateral view.

#### Remarks.

The dorsal process of 9^th^ tergite in the male of *Osmylus angustimarginatus* sp. n. is finger-like (Fig. 9a), closely resembling the condition observed in *Osmylus maoershanicola* sp. n. However, the ventral margin of the gonarcus of *Osmylus angustimarginatus* sp. n. is well sclerotized (Fig. 9b), clearly differentiating it from *Osmylus maoershanicola* sp. n. Moreover, female gonapophysis lateralis of *Osmylus angustimarginatus* sp. n. is more slender in comparison with the fusiform gonapophysis lateralis of *Osmylus maoershanicola* sp. n. (Fig. 5f).

## Supplementary Material

XML Treatment for Osmylus

XML Treatment for Osmylus maoershanicola

XML Treatment for Osmylus shaanxiensis

XML Treatment for Osmylus angustimarginatus
